# An Atypical Initial Manifestation of Systemic Lupus Erythematosus: Lupus Enteritis Accompanied by Intestinal Pseudo-Obstruction and Bilateral Hydronephroureter

**DOI:** 10.7759/cureus.50628

**Published:** 2023-12-16

**Authors:** Faiza Naeem, Mishkawt U Noor, Shabnam Batool, Saira E Anwer Khan, Muhammad Akmal

**Affiliations:** 1 Rheumatology, Shalamar Institute of Health Sciences, Lahore, PAK

**Keywords:** systemic lupus erythematosus, sle enteritis, neuropsychiatric systemic lupus erythematosus (npsle), hydronephroureter, intestinal pseudo-obstruction

## Abstract

Systemic lupus erythematosus (SLE) is a systemic, autoimmune, multisystem disease. Lupus enteritis accompanied by intestinal pseudo-obstruction (IPO) is a serious and rare initial manifestation that can lead to high mortality and morbidity in case of delay in diagnosis and treatment.

Here, we present a very complicated case of a 36-year-old female Pakistani patient with lupus enteritis accompanied by IPO and bilateral hydronephroureter. The patient had a three-month history of fever, weight loss, recurrent diarrhea, vomiting, alopecia, and photosensitivity. She had a malar and discoid rash, with signs and symptoms of IPO and neuropsychiatric lupus. Her labs revealed positive anti-nucleosome antibodies (8 U/mL), anti-Ro antibodies (100 U/mL), and anti-La antibodies (53 U/mL); equivocal anti-dsDNA antibodies (7 U/mL) and anti-Sm antibodies (7 U/mL); direct Coomb’s positive hemolytic anemia; raised C-reactive protein and erythrocyte sedimentation rate levels; low complement (C3 and C4) levels; and pyuria. IPO was evident on abdominal X-ray and CT scan. Her Systemic Lupus Erythematosus Disease Activity Index was 24, indicating severe disease flare. She was treated with intravenous methylprednisolone, hydroxychloroquine, and intravenous 500 mg cyclophosphamide. Her lab parameters and clinical mini-mental score improved, from 0/30 to 18/30. She was discharged on oral prednisolone 0.5 mg/kg/day, hydroxychloroquine, trimethoprim-sulfamethoxazole (prophylaxis for *Pneumocystis jirovecii* pneumonia), and mineral and vitamin supplements. She was followed up on the 15th day of discharge for the next dose of cyclophosphamide, and her clinical and lab parameters were normal at that time with gradual improvement in cognition.

Lupus enteritis with coexisting IPO and bilateral hydronephroureter poses a diagnostic and therapeutic challenge because of atypical and uncommon manifestations of lupus and overlapping features with intestinal tuberculosis and other inflammatory bowel conditions.

## Introduction

Systemic lupus erythematosus (SLE) is a multifactorial autoimmune disease with multisystem involvement, resulting from autoantibody production due to immune system dysfunction and activation of the complement system. It is diagnosed using the American College of Rheumatology criteria [1]. The incidence of SLE ranges from 0.6% to 2% with a female-to-male ratio of 2:1. The peak age of incidence in females is during the third to seventh decade, whereas in males it is during the fifth to seventh decade [2].

A serious and atypical initial presentation of SLE is lupus enteritis, with a high mortality rate (53%) if complicated, or if treatment is delayed [3]. Lupus enteritis was defined by the British Isles Lupus Assessment Group in 2004 as vasculitis or inflammation of the small bowel, diagnosed based on clinical features supported by suggestive imaging, with a CT scan of the abdomen as the gold standard, and/or biopsy findings [3,4].

Zhang et al. reported that the prevalence of lupus intestinal pseudo-obstruction (IPO) was 1.96%, with an in-hospital fatality rate of 7.1%. IPO presents as an initial manifestation in 57.6% of lupus patients and the rate of misdiagnosis has been reported to be 78%[5]. It is defined as the dilation of the bowel without the presence of an anatomical obstruction, with presenting signs and symptoms of nausea, vomiting, abdominal distension, and obstipation along with bowel dilation on X-ray or CT imaging [6].

SLE-IPO is strongly associated with genitourinary complications, including hydronephrosis, hydroureter, and cystitis. Approximately 60% of SLE-IPO cases have coinciding ureterohydronephrosis, which is defined as the dilation of the entire upper urinary tract, including the renal pelvicalyceal system and the ureter [7].

In this report, we describe a rare initial clinical presentation of SLE as lupus enteritis accompanied by IPO with bilateral hydronephroureter, as it presents a challenge in diagnosis and treatment.

## Case presentation

A 36-year-old Pakistani female with no comorbid factors, having five children and two neonatal deaths, reported a history of recurrent episodes of diarrhea, vomiting, generalized abdominal pain, low-grade fever, and undocumented weight loss for three months. She had developed a rash over her face and body with hair fall, oral ulcers, and photosensitivity two months before her current hospitalization. This time she presented with aggravation of gastrointestinal symptoms for the last five days with complaints of loose stools, five episodes in a day, soft in consistency, not containing blood, mucus, or pus, associated with a history of urgency; however, no history of tenesmus was reported. The patient also had generalized abdominal pain and vomiting for the last five days, with vomitus containing food particles. There was no complaint of hematemesis or melena. Later, during her current hospital stay, she developed IPO with complaints of constipation, vomiting, aggravation of abdominal pain with abdominal distension, and absolute constipation. A few days later, after worsening gastrointestinal symptoms, she developed psychosis and cognitive impairment.

She had been initially evaluated at a hospital for her gastrointestinal complaints, where intestinal tuberculosis was suspected and a workup was done, as the patient also had a history of exposure to pulmonary tuberculosis from close relatives; however, there was no history of recent travel and personal and family history of psychological disorders, with no positive family history of lupus or other autoimmune disorders. Previously, she was treated for suspected infective enterocolitis and urinary tract infection with intravenous antibiotics, metronidazole and meropenem. No record was found for administration of oral or intravenous steroids, hydroxychloroquine, or any disease-modifying anti-rheumatic drugs.

Examination on current admission

**Figure 1:**
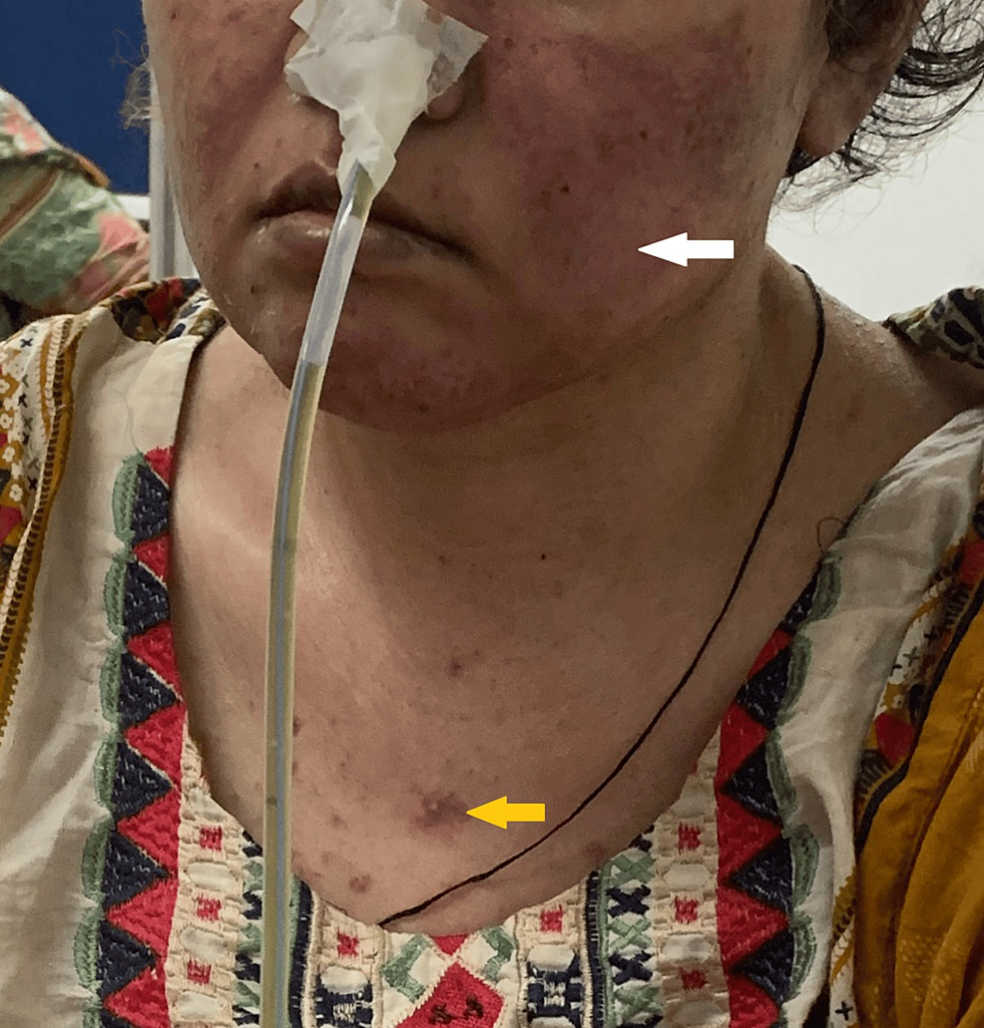
Malar rash (white arrow) and discoid rash (yellow arrow).

**Figure 2:**
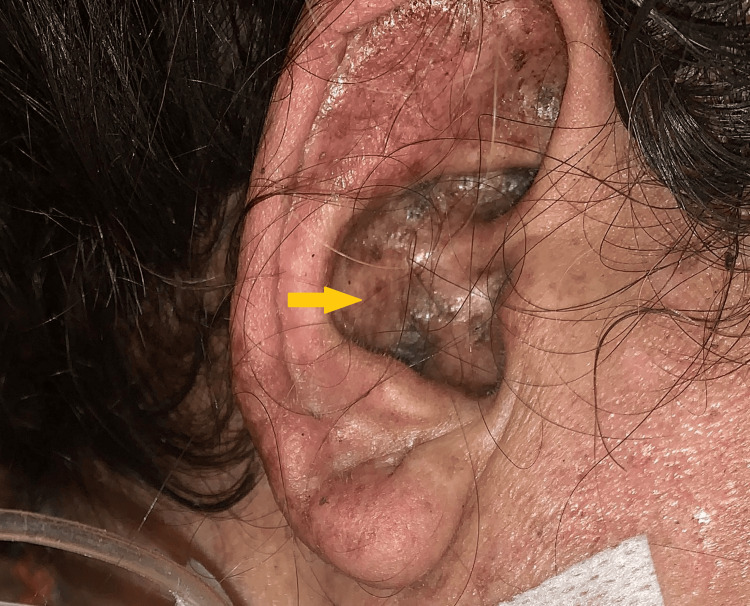
Shuster sign (yellow arrow).

Her vitals were stable except for tachycardia with normal rhythm. She had a malar rash, discoid rash, Shuster sign, alopecia, oral ulcers, and oral thrush (Figures 1, 2). The abdomen was soft with generalized mild tenderness, but no visceromegaly or ascites was seen, and gut sounds were diminished. Later, when she developed IPO, her abdomen was tense, distended, and extremely tender, with absent gut sounds. Her mini-mental score was assessed when she developed neuropsychiatric symptoms, and it was 0/30 (severe). The rest of the systemic and musculoskeletal examinations were unremarkable.

Laboratory investigations

Her recent labs showed direct Coombs-positive hemolytic anemia, hypokalemia, and raised C-reactive protein (CRP). Her extractable nuclear antigen profile (reference value U/mL: negative <6, equivocal 6-12, positive >12) showed raised anti-nucleosome antibodies (8 U/mL), equivocal anti-dsDNA antibodies (7 U/mL) and anti-Sm antibodies (7 U/mL), and positive anti-Ro antibodies (100 U/mL) and anti-La antibodies (53 U/mL).

Her previous laboratory testing revealed positive anti-nuclear antigen by enzyme-linked immunosorbent assay (ELISA), low complement (C3 and C4) levels, anemia, proteinuria, 24-hour urinary protein of 322 mg/dL (<10 mg/dl), and urine culture and sensitivity showed *Candida *and *Escherichia coli* sensitive to meropenem. However, on the current admission, she had no proteinuria. Her hepatitis B surface antigen and anti-hepatitis C virus antibodies by ELISA were negative. MRI of the brain and echocardiography were normal. The rest of the labs are shown in Table 1.

**Table 1:** Trends in laboratory parameters. ESR = erythrocyte sedimentation rate; CRP = C-reactive protein; WBC = white blood cell; RBC = red blood cell; PCV = packed cell volume; HCT = hematocrit; MCH = mean corpuscular hemoglobin; SGPT = serum glutamate pyruvate transaminase; ALT = alanine transaminase; SGOT = serum glutamic-oxaloacetic transaminase; AST = aspartate transaminase; HPF = high-power field

Labs	Reference value	30-07-23	04-08-23	10-08-23	14-08-23	19-8-23	28-08-23	7-11-23
ESR	10–20 mm/first hour	12	57	23	51	-	10	-
CRP	<5 mg/L	44	11.4	9.9	159.8	-	-	1.5
Hemoglobin	12–16 g/dL	-	10.2	10.6	9.5	7.3	11.1	11.7
WBC	(4–11 × 10^3^/µL	-	8.41	17.43	16.9	7.3	8.80	7.8
RBC	4–6 × 10^3^/µL	-	3.53	3.59	3.32	3.9	4.66	4.9
Platelet	150–450 × 10^3^/µL	-	375	118	63	139	204	406
PCV (HCT)	42–47%	-	30.9	36.6	26.8	28.3	41.5	40.1
MCH	29–32 pg	28.2	28.9	28.3	27.7	29.2	30.3	35.9
Bilirubin total	0.10–1.10 mg/dL	-	-	0.5	0.6	-	-	0.5
Bilirubin direct	0.10–0.40 mg/dL	-	-	0.5	0.5	-	-	0.3
Bilirubin indirect	0.10–0.70 mg/dL	-	-	0.1	0.1	-	-	0.2
Alkaline phosphatase	40–135 U/L	-	-	112	107	-	-	-
SGPT (ALT)	5–40 U/L	-	-	209	136	-	59	33
SGOT (AST)	5–40 U/L	57	-	147	60	-	-	35
Albumin	3.50–5 g/dL	2.4	-	2.8	2.5	-	-	3.7
Proteins total	6.30–8.30 g/dL	6.1	-	5.6	5.3	-	-	7.2
Gamma-glutamyl transferase	5–50 U/L	117	-	328	273	-	-	47
Urea	10–50 mg/dL	63	30	-	-	-	-	45
Creatinine	0.50–1.30 mg/dL	1.1	0.5	-	-	-	-	0.6
Sodium	135–145 mmol/L	132	138	136	138	-	-	-
Potassium	(3.50–5.10 mmol/L)	3.2	2.7	3.6	4.1	-	-	-
Urine complete examination	Pus cell (0–5/HPF) RBC (≤ 4/HPF); protein nil; blood nil	Pus cell 4–5/HPF; protein nil; blood nil	-	Pus cell 18–20/HPF; protein nil; RBC 20–22/HPF; blood trace	-	-	-	Pus cell 0–1; Protein nil; RBC nil; blood nil
Urine culture and sensitivity	-	-	*Candida*	-	*Pseudomonas* sensitive to meropenem and amikacin	-	-	-
Blood culture and sensitivity	-	-	-	-	*Pseudomonas* sensitive to meropenem and amikacin	-	-	-

Radiological investigation

**Figure 3:**
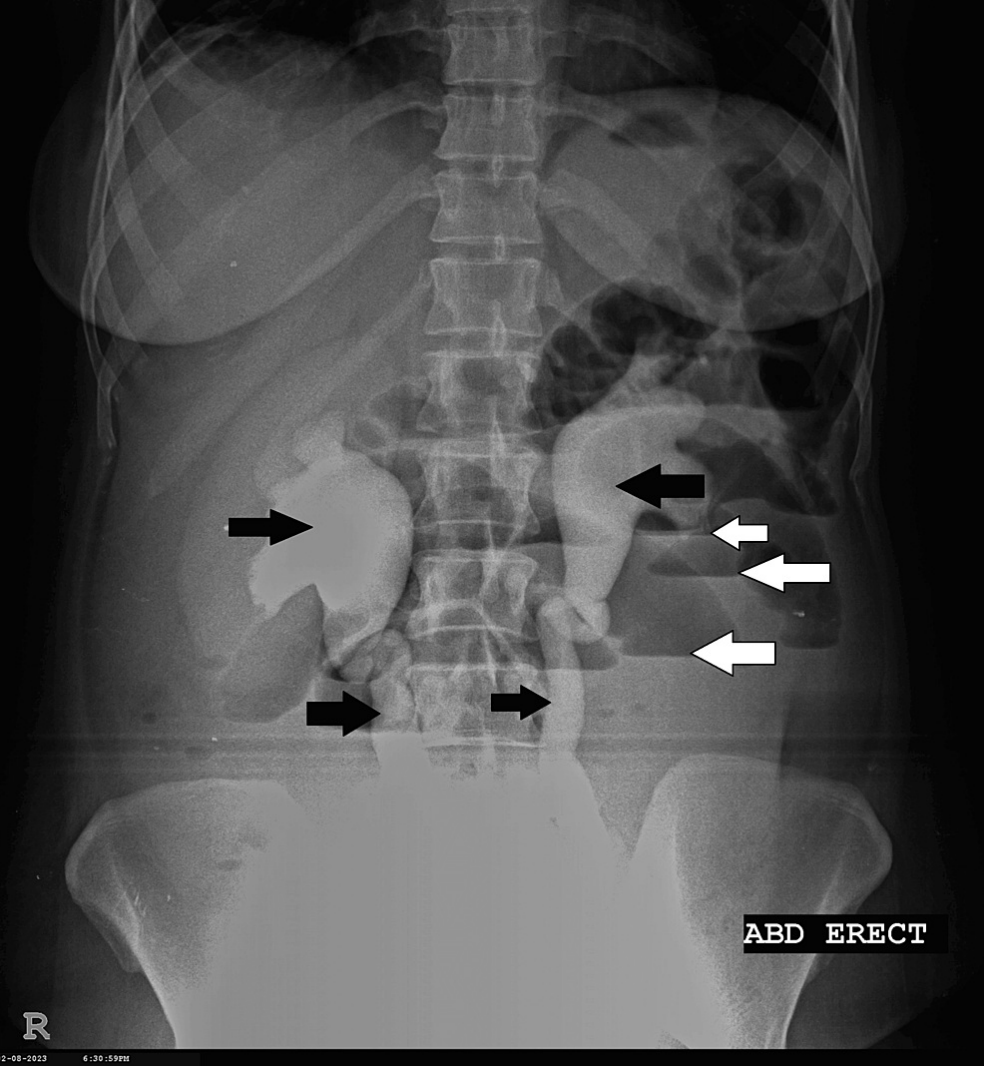
Air-fluid levels (white arrows) and bilateral hydronephroureter (black arrows).

Radiological investigations included an X-ray of the abdomen in an erect position, which showed multiple air-fluid levels and strikingly large and prominent ureters bilaterally (Figure 3). Ultrasound of the abdomen and pelvis showed multiple fluid-filled gut loops, with circumferential diffuse wall thickening, bilateral hydronephroureter, and thick-walled urinary bladder.

**Figure 4:**
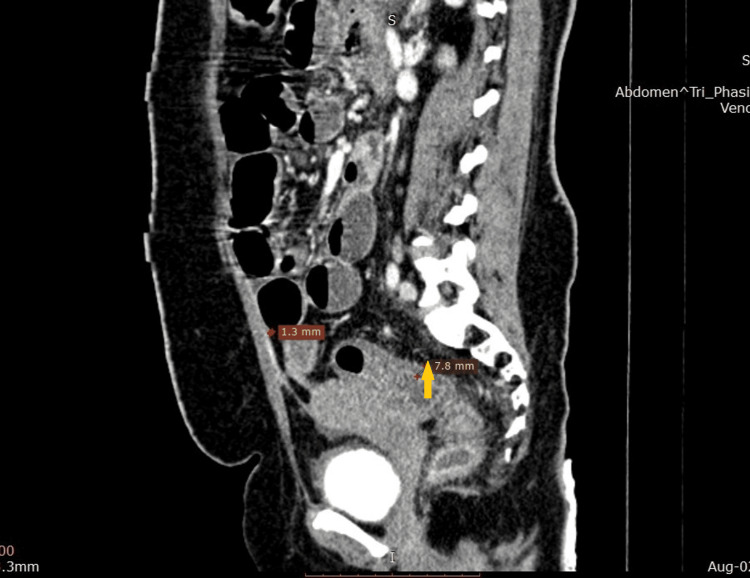
CT of the abdomen showing bowel wall thickening (yellow arrow).

**Figure 5:**
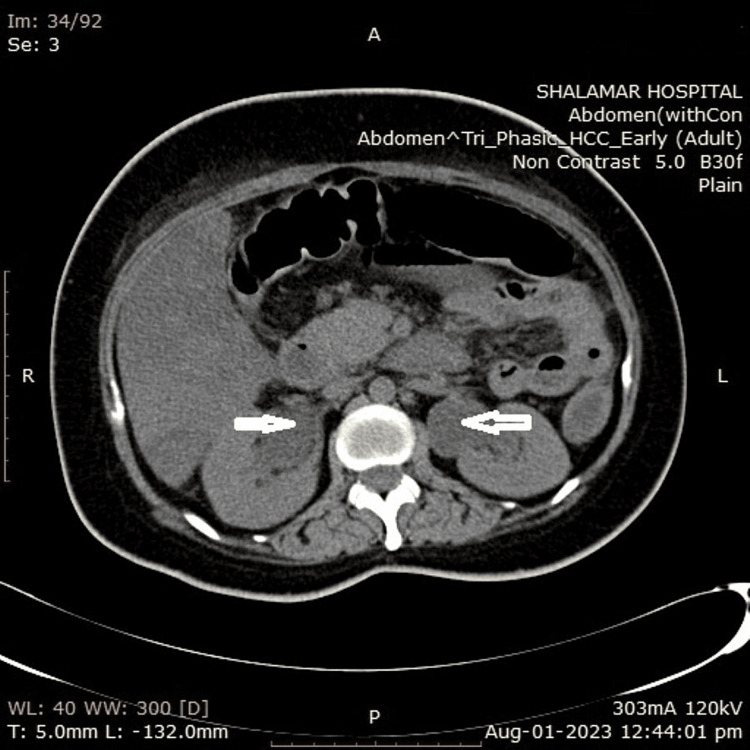
CT of the abdomen showing bilateral hydronephroureter (white arrows).

**Figure 6:**
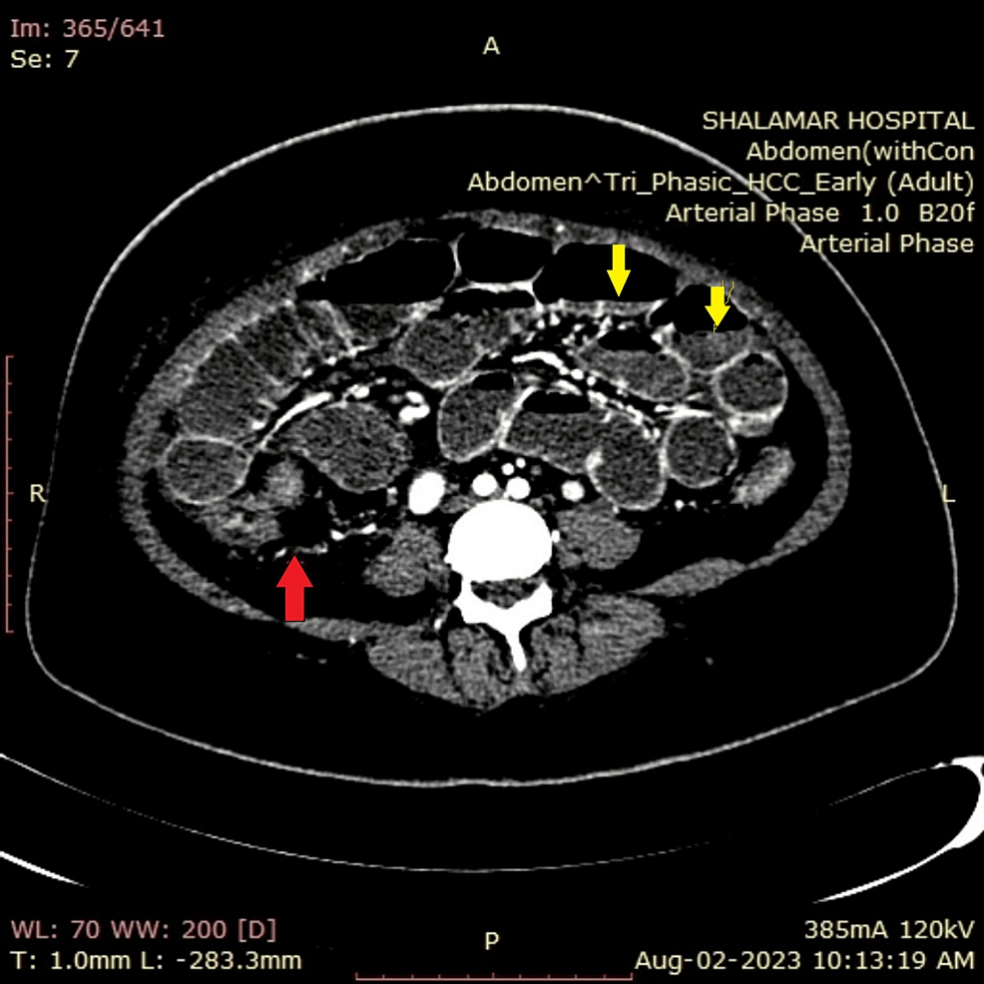
CT angiogram showing decreased vascular enhancement (red arrow) and air-fluid levels (yellow arrows).

CT of the abdomen further confirmed these findings and revealed diffuse wall thickening of the large bowel mainly along the sigmoid colon with proximal dilation of the small bowel, as well as mild reactive ascites (Figure 4), bilateral hydronephroureter along with urinary bladder wall thickening (Figure 5). CT angiography of the abdomen with contrast suggested vasculitis secondary to SLE with resultant ischemic colitis and ileitis (Figure 6). Sigmoidoscopy and biopsy were also done, which were normal. 

Management

Treatment was started on the lines of active lupus, the Systemic Lupus Erythematosus Disease Activity Index (SLEDAI) was 24 (severe flare) along with the treatment of urinary tract infection and suspected lupus/infective enterocolitis. The patient was kept nil per oral (NPO), and a nasogastric tube was passed for gastric decompression. The patient was catheterized to monitor urine output, and a central venous catheter was also passed through which partial parenteral nutrition was started. Intravenous methylprednisolone 125 mg was administered twice a day for a total dose of 3,500 mg administered during her current admission. Hydroxychloroquine 5 mg/kg/day was administered daily. Continuous intravenous fluids were administered along with intravenous potassium and intravenous albumin replacement. Her central venous pressure was checked regularly to prevent fluid overload. Moreover, injection meropenem 1 g intravenous eight hourly, tablet fluconazole 100 g once a day with monitoring of liver function tests and creatinine, and topical treatment for rash were also given. The urologist advised conservative management with bladder catheterization for bilateral hydronephroureter. For the management of psychosis, haloperidol 5 mg was administered intravenously as needed. After the urinary tract infection and fever were resolved, injection cyclophosphamide 500 mg was administered intravenously. Tablet trimethoprim-sulfamethoxazole double strength was given on alternate days for prophylaxis of pneumocystis jirovecii. A renal biopsy could not be done as she was sick at the time of admission and later developed nosocomial sepsis.

Outcome and follow-up

A few days after she was administered cyclophosphamide, she developed a spiking fever of 102-103°F with rigors and chills, raising the suspicion of nosocomial infection. All her indwelling catheters were removed and sent for cultures. Blood culture and sensitivity showed *Pseudomonas aeruginosa*, which was sensitive to amikacin. Her central venous catheter tip and Foley catheter tip culture and sensitivity showed *Candida *infection, for which fluconazole was already being administered.

**Figure 7:**
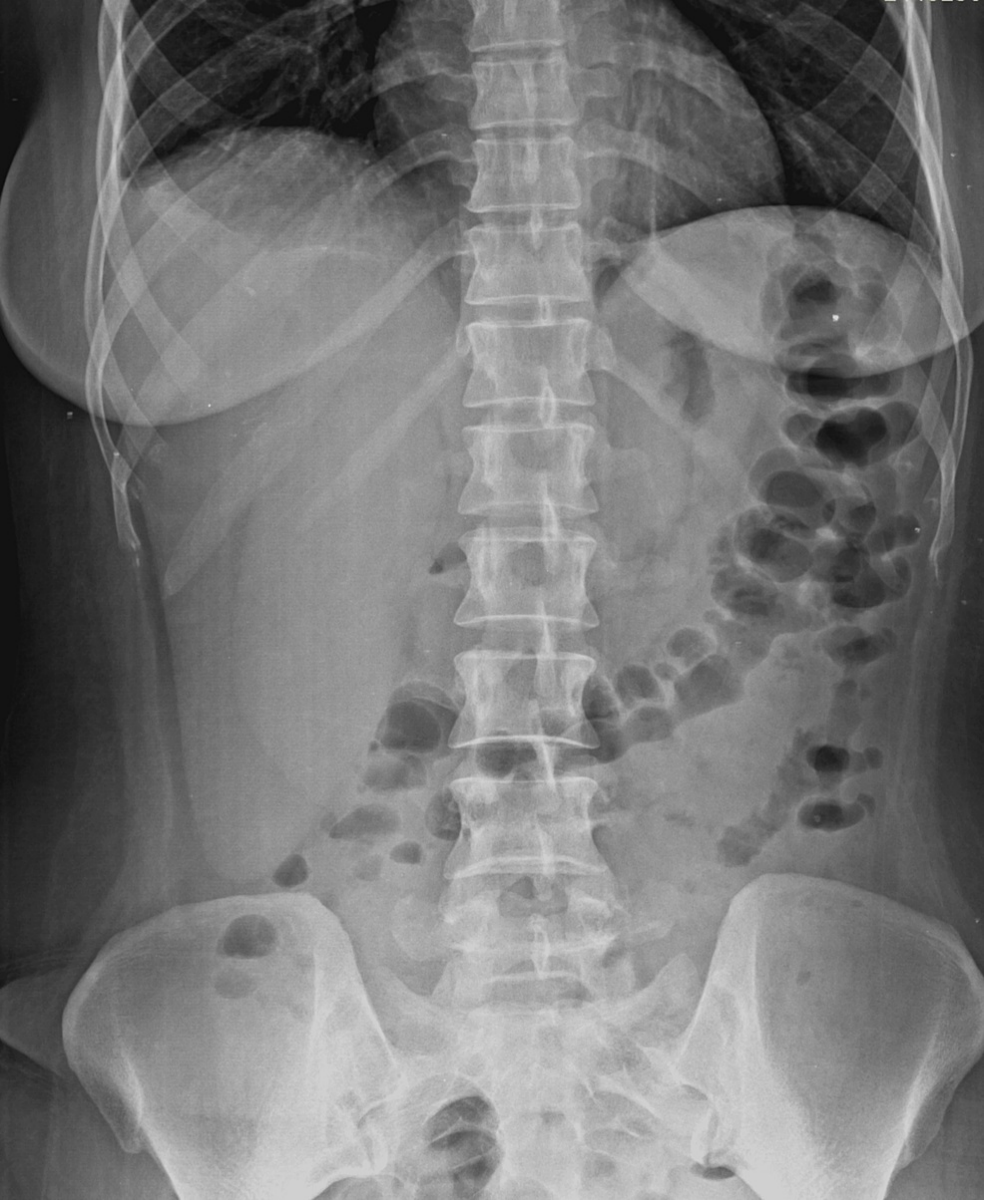
Absence of bilateral hydronephroureter and air-fluid levels.

After administering intravenous amikacin, she became afebrile, her condition improved clinically, and laboratory parameters also improved. At the time of discharge, after 20 days of hospitalization, her gastrointestinal symptoms had improved, with regular bowel movements, and improvement was seen in all clinical and radiological parameters. The absence of bilateral hydronephroureter was seen on a repeating abdominal X-ray (Figure 7). The mini-mental score improved from 0/30 (severe cognitive impairment) to 18/30 (moderate cognitive impairment), SLEDAI was not calculated due to the absence of repeated compliments and anti-DsDNA antibodies due to financial constraints.

She was discharged on oral prednisolone 0.5 mg/kg/day, along with hydroxychloroquine, trimethoprim-sulfamethoxazole, and mineral and vitamin supplements, and was called for follow-up on the 15th day of discharge for the next dose of cyclophosphamide. Now she is on regular follow-ups and currently has no gastrointestinal symptoms and progressive improvement in cognition. We plan to start mycophenolate mofetil after completing six fortnightly pulses of 500 mg cyclophosphamide over three months (Euro lupus protocol). The patient remains tolerant and adherent to current therapy and no adverse event has been observed on her follow-up visits.

## Discussion

Lupus enteritis with IPO presents a diagnostic and therapeutic dilemma, as gastrointestinal manifestations in this case mimicked infectious enterocolitis, particularly intestinal tuberculosis, Crohn’s disease, and drug-induced colitis. Non-steroidal anti-inflammatory drugs, corticosteroids, and antibiotics have been employed for the management of this condition [8-10]. This patient was being treated previously, before the development of a lupus rash, with suspicion of infectious enterocolitis/intestinal tuberculosis.

Although the pathogenesis of lupus enteritis is poorly understood, immune complex deposition in the bowel wall with complement activation might be the driving force, with the involvement of the jejunum and ileum most commonly followed by the involvement of the colon, duodenum, and rectum [11]. Patients with large intestine-dominant lupus enteritis have hydroureter and bladder wall thickening, which are complications of IPO [12].

Among patients with SLE-IPO, approximately two-thirds have positive anti-SS-A/Ro antibodies, hypocomplementemia, hypoalbuminemia, elevated CRP, and polyureterectasis [5,13]. Abdominal X-rays may show multiple air-fluid levels and enlarged small intestine segments [14]. CT abdomen findings of lupus enteritis include engorgement or increased number of mesenteric vessels (“comb sign”) [11], bowel wall thickening and enhancement (“target sign”), and increased attenuation of mesenteric fat. These imaging findings along with gut wall ischemia and enhancement can also be seen in SLE-IPO [15]. Our patient had similar lab and radiological findings with raised CRP, hypocomplementemia, hypoalbuminemia, high-titer anti-Ro antibodies, air-fluid levels on X-ray with bilateral hydronephroureter, gut and bladder wall thickening, ischemic colitis, and ileitis.

Previous case reports support the treatment strategies of using intravenous methylprednisolone at a dose of 250 mg to 1 g per day, followed by prednisolone 0.5 mg/kg/day, bowel rest, hydration, electrolyte repletion, and parenteral nutrition. Hydroxychloroquine, cyclophosphamide, cyclosporin, mycophenolate mofetil, and intravenous immunoglobulins were administered as steroid-sparing agents. The patient showed good immediate response to the above treatment regimens and maintained remission with a low incidence of relapse [16,17]. If IPO is not treated, the smooth muscle layer can become atrophic and fibrotic and will no longer be reversible with immunosuppression [18].

In our patient, improvement of gastrointestinal symptoms, hydronephroureter, and cognitive function were observed. We planned to start mycophenolate mofetil after completing six fortnightly pulses of 500 mg cyclophosphamide over three months (the Euro lupus protocol of cyclophosphamide) [19].

Our patient was managed successfully with the help of a multidisciplinary approach. All relevant investigations and treatment were performed without any delay. Because of financial constraints, few lab tests could not be repeated to calculate the clinical response.

## Conclusions

Lupus enteritis with coexisting IPO and bilateral hydronephroureter poses a diagnostic and therapeutic challenge because of atypical and uncommon manifestation of lupus and overlapping features with intestinal tuberculosis and other inflammatory bowel conditions. Particularly, if gastrointestinal symptoms occur before the typical features of SLE appear, the index of suspicion for lupus enteritis and SLE-IPO should be high in patients with combined gastrointestinal and genitourinary symptoms. Prompt diagnosis and treatment can prevent morbidity and mortality and avoid invasive procedures for hydronephroureter and IPO.

## References

[1] Dall’Era M (2021). Systemic lupus erythematosus. Current Diagnosis & Treatment Rheumatology.

[2] Rees F, Doherty M, Grainge MJ, Lanyon P, Zhang W (2017). The worldwide incidence and prevalence of systemic lupus erythematosus: a systematic review of epidemiological studies. Rheumatology (Oxford).

[3] Mushtaq H, Razzaque S, Ahmed K (2018). Lupus enteritis: an atypical initial presentation of systemic lupus erythematosus. J Coll Physicians Surg Pak.

[4] Smith LW, Petri M (2013). Lupus enteritis: an uncommon manifestation of systemic lupus erythematosus. J Clin Rheumatol.

[5] Zhang L, Xu D, Yang H (2016). Clinical features, morbidity, and risk factors of intestinal pseudo-obstruction in systemic lupus erythematosus: a retrospective case-control study. J Rheumatol.

[6] Coulie B, Camilleri M (1999). Intestinal pseudo-obstruction. Annu Rev Med.

[7] Adler BL, Timlin H, Birnbaum J (2019). Lupus intestinal pseudo-obstruction and hydronephrosis: case report. Medicine (Baltimore).

[8] Wu P, Zeng J, Yang L (2022). Case report: vesicorectal fistula caused by intestinal tuberculosis complicated with systemic lupus erythematosus. Infect Drug Resist.

[9] Zhu XL, Xu XM, Chen S, Wang QM, Zhang KG (2019). Lupus enteritis masquerading as Crohn's disease. BMC Gastroenterol.

[10] Hamdeh S, Micic D, Hanauer S (2021). Drug-induced colitis. Clin Gastroenterol Hepatol.

[11] Janssens P, Arnaud L, Galicier L (2013). Lupus enteritis: from clinical findings to therapeutic management. Orphanet J Rare Dis.

[12] Maruyama A, Nagashima T, Iwamoto M, Minota S (2018). Clinical characteristics of lupus enteritis in Japanese patients: the large intestine-dominant type has features of intestinal pseudo-obstruction. Lupus.

[13] Huang Q, Lai W, Yuan C (2016). Predictors of intestinal pseudo-obstruction in systemic lupus erythematosus complicated by digestive manifestations: data from a Southern China lupus cohort. Lupus.

[14] Chng HH, Tan BE, Teh CL, Lian TY (2010). Major gastrointestinal manifestations in lupus patients in Asia: lupus enteritis, intestinal pseudo-obstruction, and protein-losing gastroenteropathy. Lupus.

[15] Huang YT, Chung TW, Wang JJ (2017). Target sign in lupus enteritis. QJM.

[16] Oh DJ, Yang JN, Lim YJ, Kang JH, Park JH, Kim MY (2015). Intestinal pseudo-obstruction as an initial manifestation of systemic lupus erythematosus. Intest Res.

[17] García López CA, Laredo-Sánchez F, Malagón-Rangel J, Flores-Padilla MG, Nellen-Hummel H (2014). Intestinal pseudo-obstruction in patients with systemic lupus erythematosus: a real diagnostic challenge. World J Gastroenterol.

[18] Park FD, Lee JK, Madduri GD, Ghosh P (2009). Generalized megaviscera of lupus: refractory intestinal pseudo-obstruction, ureterohydronephrosis and megacholedochus. World J Gastroenterol.

[19] Ajili F, Ben Ariba Y, Sayehi S (2013). AB0369 Evaluation of the eurolupus protocol in lupus nephritis treatment in an internal medicine department. Ann Rheum Dis.

